# The Clinical Utility of Circulating Epstein-Barr Virus DNA Concentrations in NK/T-Cell Lymphoma: A Meta-Analysis

**DOI:** 10.1155/2018/1961058

**Published:** 2018-11-18

**Authors:** Ze-Long Liu, Xi-Wen Bi, Pan-Pan Liu, De-Xin Lei, Wen-Qi Jiang, Yi Xia

**Affiliations:** Department of Medical Oncology, Sun Yat-sen University Cancer Center, State Key Laboratory of Oncology in South China, Collaborative Innovation Center of Cancer Medicine, Guangzhou, China

## Abstract

**Background:**

Circulating Epstein-Barr virus (EBV) DNA concentrations were reported to have prognostic value for NK/T-cell lymphoma patients in limited small-scale studies. In this study, we aimed to evaluate the clinical utility of circulating EBV-DNA concentrations to a large sample of NK/T-cell lymphoma patients.

**Methods:**

We conducted this meta-analysis, which included a total of 15 prospective and retrospective comparable studies to assess the association between pretreatment EBV-DNA (pre-DNA), posttreatment EBV-DNA (post-DNA), and clinical outcomes of NK/T-cell lymphoma patients. We chose overall survival (OS) as the primary endpoint and progression-free survival (PFS), complete response (CR), and overall response rate (ORR) as secondary endpoints.

**Results:**

High pre-DNA and detectable post-DNA were both significantly correlated with poorer OS in NK/T-cell lymphoma patients (*P* < 0.05), with hazard radios (HRs) equal to 3.45 and 2.30, respectively. High pre-DNA and detectable post-DNA also predicted poorer PFS. Additionally, high pre-DNA was found to be significantly correlated with both worse CR and ORR, which indicated worse treatment response.

**Conclusion:**

Circulating EBV-DNA concentrations provides prognostic values of survival and treatment response in NK/T-cell lymphoma patients.

## 1. Introduction

As reported, NK/T-cell lymphoma constituted 10.4–11.8% of T-cell lymphomas [1]. Its incidence is relatively higher in Asia than in other regions and accounts for 22.4% of T-cell lymphomas [1]. NK/T-cell lymphoma is more common in males than females with a ratio of 2 : 1, and the median age at its diagnosis is 49 years [2]. According to current published studies, 60% to 90% of cases are localized to the nasal and upper airway region (nasal NK/T-cell lymphoma) [2]. Nowadays, patients with localized nasal NK/T-cell lymphoma are recommended treatment with concurrent chemoradiotherapy (CCRT), and their 5-year overall survival (OS) has reached to approximately 70% [3]. For patients with advanced or relapsed/refractory NK/T-cell lymphoma, the efficacy of chemotherapy regimens containing l-asparaginase has been confirmed in many studies. However, furthermore, researches should be performed for improvements in the individualized treatment of NK/T-cell lymphoma patients.

Circulating EBV-DNA concentrations correlated positively with disease stage and also exhibited prognostic values in NK/T-cell lymphoma. Quantitative detection of EBV-DNA in plasma is extensively used clinically since it is valuable for monitoring treatment response and evaluating prognosis [4]. Kim et al. reported a prognostic model named PINK-E for NK/T-cell lymphoma patients after non-anthracycline-based treatment, which included detectable circulating EBV-DNA before treatments as an independent prognostic factor [5]. They found that stratification of patients based on this prognostic model showed a significant association with OS and PFS in their cohorts [5]. In 2015, Wang et al. reported that posttreatment plasma EBV-DNA positivity predicts early relapse and poor prognosis for patients with extranodal NK/T-cell lymphoma (ENKL) in the era of asparaginase [6]. However, procedures of quantification of EBV-DNA have not been standardized, and so the cut-off value of high titer circulating EBV-DNA has not been decided. On the other hand, the published data are inadequate due to current limited studies of small-scale population. So we conducted this meta-analysis in a large-scale population to explore the prognostic value of quantification of EBV-DNA in peripheral blood before and after treatment in NK/T-cell lymphoma patients.

## 2. Materials and Methods

### 2.1. Ethics Statement

This study was conducted in accordance with the principles of the Helsinki Declaration with the approval from the Academic Committee of Sun Yat-Sen University Cancer Center. The data in this study were obtained from published studies, which all contained informed consent.

### 2.2. Search Strategy

A comprehensive search of PubMed, Web of Science, EMBASE, and the Cochrane Library was conducted independently by two investigators (ZL.L. and XW.B.). The search strategy was based on combinations of “(EBV DNA) OR (Epstein-Barr virus DNA) OR (Epstein-Barr viral DNA) OR (EBV deoxyribonucleic acid) OR (Epstein Barr virus deoxyribonucleic acid) OR (Epstein-Barr viral deoxyribonucleic acid)” and “(NK/T cell lymphoma) OR (natural killer/T cell lymphoma) OR (angiocentric lymphoma)” in the [Title/Abstract]. In order to find more eligible studies for our research, reference literatures of selected studies and relevant published systematic reviews were also searched separately. The latest date of the search was June 7, 2018.

### 2.3. Outcomes

We chose OS and PFS as our primary endpoints and reported them as unadjusted HRs. Our secondary endpoints were CR and ORR, which were reported as RRs. OS was defined as the period of time from the date of treatment to the date of death or the date of the last follow-up visit. PFS was calculated from the end date of the initial treatment to the date of the first local or distance relapse or to the date of the last follow-up or death. CR, partial response (PR), and ORR were defined according to revised response criteria for malignant lymphoma (2007) [7].

### 2.4. Inclusion Criteria and Exclusion Criteria

Inclusion criteria of studies were listed as follows: (1) NK/T-cell lymphoma patients with a confirmed pathological diagnosis and (2) contained at least one of the primary endpoints (OS and PFS) or secondary endpoints (CR and ORR). For OS and PFS, HRs and corresponding 95% confidence intervals (CIs) should be reported directly or could be indirectly calculated from other types of data, such as survival curves. Systematic reviews, abstracts, comments, editorials, case reports, animal model studies, and single-arm studies were all excluded in this study. If there were several literatures originating from the same population, only the most recent and complete study was included. The inclusion and exclusion of each study was independently assessed by two investigators (ZL.L. and XW.B.), and discussions were proceeded to solve disagreements.

### 2.5. Data Extraction and Quality Assessment

Two investigators (ZL.L. and XW.B.) extracted and recorded the data independently in a predesigned table and reached a consensus for all information. The extracted data included the first author, year of publication, study design, inclusion period, number of patients, age, Ann Arbor stage, sample, cut-off value of EBV-DNA (both of pre-DNA and post-DNA), treatment strategy, survival outcomes, and follow-up time. Two investigators (ZL.L. and XW.B.) made an independent evaluation on the quality of each study, through use of the Newcastle–Ottawa quality assessment scale (NOS) for observational studies [8]. A third investigator (PP.L.) was consulted to solve any disagreements. The total quality scores ranged from 0 to 9 points. The results of the quality assessment of all included studies are shown in Table 1.

### 2.6. Statistical Analysis

Unadjusted HRs and corresponding 95% CIs were extracted from included studies for survival analysis. For studies wherein unadjusted HRs and corresponding 95% CIs were unavailable, we used other types of statistics or Kaplan-Meier survival curves to calculate the HRs according to the methods reported by Parmar and colleagues [19]. For CR and ORR, we used the RRs and corresponding 95% CIs as measures of outcome. Chi^2^ test and *I* ^2^ statistic were applied to evaluate the heterogeneity among eligible studies. If *P* value was >0.10 in chi^2^ test or *I* ^2^ value was <50%, we considered no statistically significant heterogeneity among studies, and then the fixed-effects model was utilized for analysis; otherwise, the random-effects model was utilized [20]. We used the inverse variance method to analyze HR data in this meta-analysis. Subgroup analyses and sensitivity analysis were conducted to find the potential sources of heterogeneity. Potential publication bias was assessed through funnel plots and Begg's tests performed in Stata 12.0 (StataCorp, College Station, TX, USA). *P* < 0.05 was defined to indicate significant publication bias. The Review Manager 5.3 (Cochrane Collaboration, Oxford, UK) was used for the meta-analysis and generating forest plots.

## 3. Results

### 3.1. Characteristics of Studies

**Figure 1:**
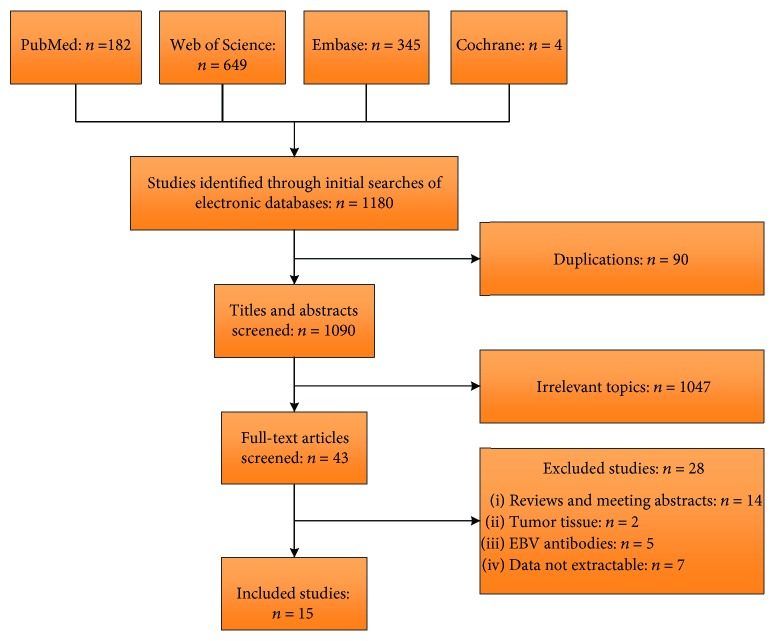
The flow chart of selection of the included studies.

A total of 1180 literatures were retrieved in our initial search, of which fifteen eligible studies were included eventually [6]. The flow chart of inclusion of the studies in this meta-analysis is presented in Figure 1. Characteristics of the 15 eligible studies are exhibited in Table 2. No RCT was available for our study. The included studies consisted of eight prospective studies [6] and seven retrospective studies [21].

Ito et al. discussed the relationship between pre-DNA both in whole blood and plasma and prognosis of patients with NK/T-cell lymphoma [22]. This study actually had two relatively independent groups of results according to our design, and we regarded it as “two studies” in the following analysis. In this study, the result of pre-DNA in plasma was represented with the study ID of “Y Ito 2012 (1)” and the result of pre-DNA in whole blood was represented with the study ID of “Y Ito 2012 (2)”.

Twelve studies reported the relationship between pre-DNA and clinical outcomes [6], whereas five reported the relationship between post-DNA and clinical outcomes [6]. OS, PFS, CR, and ORR data were extractable in eleven studies, five studies, three studies, and five studies, respectively, for comparing clinical outcomes based on pre-DNA. OS and PFS data for studies of correlation between post-DNA and clinical outcomes were available in only five studies and four studies, respectively. The results of this meta-analysis are summarized in Table 3.

### 3.2. Pre-DNA and Clinical Outcomes

**Figure 2:**
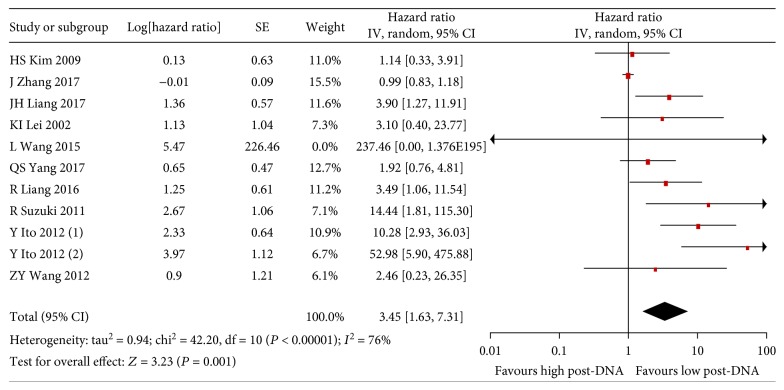
Forest plot of the analysis for pre-DNA-associated OS.

Based on pooled analysis of OS data from 11 studies, high pre-DNA levels were found to be significantly correlated with poorer OS in NK/T-cell lymphoma patients (HR 3.45, 95% CI 1.63–7.31, *P* = 0.001) (Figure 2). Since there was statistically significant heterogeneity among these studies (*I* ^2^:76%, *P* < 0.00001), we subsequently performed subgroup analyses and sensitivity analysis. The HR of PFS for patients with high pre-DNA levels was 2.29 (Supplementary Figure S1). The RRs of CR and ORR were 1.45 and 1.57, respectively (Supplementary Figures S2 and S3).

### 3.3. Post-DNA and Clinical Outcomes

**Figure 3:**
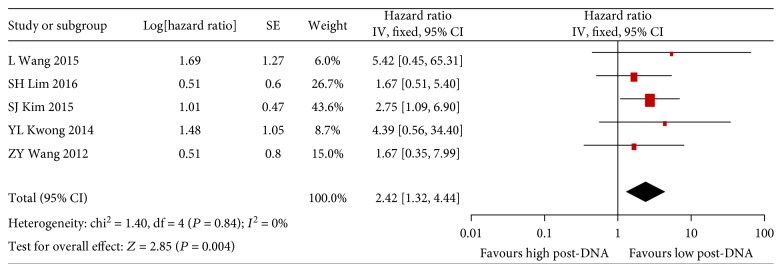
Forest plot of the analysis for post-DNA-associated OS.

It is found that detectable post-DNA was significantly associated with poorer OS of NK/T-cell lymphoma patients as high pre-DNA (HR 2.42, 95% CI 1.32–4.44, *P* = 0.004) (Figure 3). Heterogeneity was undetected between the five included studies (*I* ^2^: 0%, *P* = 0.84). Subgroup analyses were performed subsequently. Similarly, the HR was 2.36 (95% CI 1.40–3.98, *P* = 0.001) for PFS in NK/T-cell lymphoma patients with detectable post-DNA compared with those without detectable post-DNA (Supplementary Figure S4).

### 3.4. Subgroup Analysis and Sensitivity Analysis

As presented in our results of data synthesis, significant heterogeneity existed between studies in the analysis of association between pre-DNA and OS. To investigate potential sources, we performed subgroup analysis and found that the heterogeneity in most of subgroups decreased (Supplementary Tables 1 and 2). We subsequently conducted a sensitivity analysis to furthermore investigate sources of the heterogeneity and found that a particular study was closely related to the heterogeneity [23]. The authors declared that they had excluded NK/T-cell lymphoma patients with brain invasion or distant metastasis in their research. These patients usually have high titer of circulating EBV-DNA concentrations and worse prognosis. Exclusion of these patients would cause an obvious selection bias according to our study. However, the analysis result remained constant after exclusion of this study (HR 3.59, 95% CI 2.28–5.66, *P* = 0.08), and the heterogeneity was eliminated (Supplementary Figure S5).

### 3.5. Publication Bias

We conducted Begg's test for publication bias of 11 studies [6] that reported association between pre-DNA and OS, which indicated there was no significant publication bias (*P* = 0.755) (Supplementary Figure S6).

## 4. Discussion

EBV infection was found to be closely associated with the occurrence, progression, and prognosis of NK/T-cell lymphoma according to current studies [24]. However, the underlying mechanism by which EBV promoted carcinogenesis remained to be elucidated. In recent studies, EBV has been observed to promote Th2-skewed T-cell responses and upregulate the expression of immune checkpoint ligand PD-L1 to influence the tumor microenvironment [24]. Anti-PD-1 and other immunological checkpoint inhibitors are recently reported to be effective for relapsed/refractory ENKL, which open up new prospects for individualized treatments of NK/T-cell lymphoma patients [25].

Given the crucial roles of EBV infection in NK/T-cell lymphoma, a number of researches have been done to explore the prognostic value of circulating EBV-DNA concentrations. However, the conclusions from these studies were somewhat controversial possibly due to limited sample size in each of them. We thus performed this meta-analysis to expand the sample size and aimed to provide a better understanding of the prognostic value of circulating EBV-DNA concentrations in patients with NK/T-cell lymphoma.

In this review, 15 studies were included according to the inclusion criteria. Circulating EBV-DNA concentrations was detected by polymerase chain reaction (PCR) technology in all included studies, using peripheral blood sample, either plasma or whole blood. DNA from samples was extracted, and EBV-DNA copy number was quantified by real-time PCR based on amplification of the EBNA1 gene according to these studies. The concentration was then calculated and expressed in copies/ml.

We observed that the pooled HR for all studies on patients with high levels of pre-DNA was greater than 1, indicating that high pre-DNA was significantly correlated with poorer OS and PFS in NK/T-cell lymphoma patients. High pre-DNA was also found to be significantly correlated with both worse CR and ORR, which indicated worse treatment response and clinical outcomes. The pooled HR for all studies on detectable post-DNA patients was found to be greater than 1, which indicated that detectable post-DNA predicted poorer OS and PFS in NK/T-cell lymphoma patients.

As mentioned before, heterogeneity was detected in this meta-analysis, which might have originated from many sources such as number of patients, sample of quantification of EBV-DNA, Ann Arbor stage, and cut-off value. We remarkably reduced the heterogeneity after excluding one single study through sensitivity analysis. The analysis for publication bias showed that there was no evidence of significant publication bias in our meta-analysis.

In summary, our meta-analysis, for the first time, confirmed that both high pre-DNA and detectable post-DNA were significantly associated with poorer OS and PFS in patients with NK/T-cell lymphoma.

It is worth noting that the results of this meta-analysis must be interpreted cautiously due to some unavoidable limitations. Firstly, the included 15 studies were all nonrandomized controlled trials with relatively small sample sizes. For instance, only 13 patients were included in the study by Liang et al. Secondly, publication and reporting bias were unavoidable because our analysis was based on data extracted from published literatures rather than original individual patient data. Thus, it is impossible for us to include all data of endpoints and basic information for each study. The meta-analysis was based on the assumption that differences between the results of various studies were caused by chance.

Quality of the included studies varied from each other. To reduce the risk of bias, two investigators independently extracted data from included studies and we scored the quality of the studies. Additionally, as presented in the Results, the heterogeneity was statistically significant between studies in the analysis of correlation between both pre-DNA and post-DNA and OS. Subgroup analysis and sensitivity analysis were performed to explore the potential sources of the heterogeneity, and results did not change after sensitivity analysis, which indicated the robustness of our conclusions. Systematic analysis that included RCTs should be performed to confirm the prognostic values and the cut-off value of circulating EBV-DNA concentrations in NK/T-cell lymphoma patients.

## 5. Conclusion

Our meta-analysis revealed that high pre-DNA and detectable post-DNA were both significantly associated with poorer OS and PFS of NK/T-cell lymphoma patients. High pre-DNA was also significantly associated with lower CR and ORR. Considering the intrinsic limitations of the included studies, well-designed RCTs are required to confirm the findings of this study and to develop the individualized treatment strategies for NK/T-cell lymphoma patients in the future.

## Figures and Tables

**Table 1:** Quality assessment by Newcastle–Ottawa scale.

Study	Representativeness of the exposed cohort	Selection of the nonexposed cohort	Ascertainment of exposure	Outcome of interest was not present at start of study	Comparability of cohorts on the basis of the design or analysis	Assessment of outcome	Follow-up was long enough	Adequacy of follow-up of cohorts	Total quality score
Lei et al. [21]	1	1	1	1	0	1	1	0	6
Kim et al. [9]	1	1	1	1	0	1	1	0	6
Suzuki et al. [10]	1	1	1	1	0	1	1	1	7
Wang et al. [11]	1	1	1	1	2	1	1	0	8
Y Ito 2012 (1)	1	1	1	1	0	1	1	1	7
Y Ito 2012 (2)	1	1	1	1	0	1	1	1	7
Kwong et al. [12]	1	1	1	1	0	1	1	0	6
Liu et al. [13]	1	1	1	1	2	1	1	1	9
Kim et al. [14]	1	1	1	1	0	1	1	1	7
Wang et al. [6]	1	1	1	1	2	1	1	0	8
Lim et al. [15]	1	1	1	1	0	1	1	0	6
Liang et al. [16]	1	1	1	1	0	1	1	0	6
Yang et al. [17]	1	1	1	1	0	1	1	1	7
Zhang et al. [23]	1	1	1	1	0	1	1	1	7
Liang et al. [18]	1	1	1	1	0	1	1	1	7

**Table 2:** Characteristics of the included studies.

Study	Design	Inclusion period	*N*	Median or mean age	Ann Arbor stage	Sample	Cut-off value		Treatment strategy	Extractable survival outcomes	Median follow-up (months)
							Pre-DNA	Post-DNA			
Lei et al. [21]	R	1995–2001	26	61y^a^	I–IV	Plasma	600	—	CT/RT/CT → RT	OS	13 (4.5–24.3)
Kim et al. [9]	R	2004–2007	47	48.5y^a^	I–IV	Whole blood	Median	—	NR	OS	NR
Suzuki et al. [10]	P	2004–2007	32	55y^a^	I–IV	Plasma	1000	—	CT/RT/CCRT/RT → CT	OS	NR
Wang et al. [11]	P	2007–2009	69	39y^a^	I–II	Plasma	500	0	RT/RT → CT	OS, PFS	32
Y Ito 2012 (1)	P	NR	26	46.5y^a^	NR	Plasma	10E5	—	CT	OS, CR, and ORR	NR
Y Ito 2012 (2)	P	NR	26	46.5y^a^	NR	Whole blood	1000	—	CT	OS, CR, and ORR	NR
Kwong et al. [12]	P	2005–2012	54	52.5y^a^	I–IV	Plasma	—	0	CT	OS	NR
Liu et al. [13]	R	2011–2014	109	40y^a^	I–IV	Plasma	500	—	NR	PFS, ORR	NR
Kim et al. [14]	R	2005–2013	102	48y^a^	I–IV	Whole blood	—	0	CT/CCRT/CT → RT	OS, PFS	47.2 (30–65.5)
Wang et al. [6]	P	2008–2014	68	47y^a^	I–II	Plasma	0	0	CT → RT	OS, PFS, and CR	32 (2–76)
Lim et al. [15]	R	2009–2014	27	44y^a^	I–IV	Whole blood	—	0	CCRT	OS, PFS	36.9 (1.6–75.4)
Liang et al. [15]	R	2007–2012	13	43.5y^a^	I–IV	Plasma	Median	—	CT/RT/CCRT/CT → RT/RT → CT	OS	NR
Yang et al. [17]	R	2006–2016	81	41y^a^	I–IV	Plasma	500	—	CT/CT → RT/RT → CT	OS,PFS	21 (1–123)
Zhang et al. [23]	P	2010–2014	85	38.66y^b^	I–IV	Plasma	0	—	CT → RT	OS, PFS, CR, and ORR	NR
Liang et al. [18]	P	2010–2015	32	48y^a^	III–IV	Whole blood	5000	—	CT	OS, PFS, and ORR	NR

R: retrospective study; P: prospective study; NR: not reported; *N*: number of participants; pre-DNA: pretreatment EBV-DNA; post-DNA: posttreatment EBV-DNA; CT: chemotherapy; RT: radiotherapy; CCRT: concurrent chemoradiotherapy; OS: overall survival; PFS: progression-free survival; CR: complete response; ORR: overall response rate. ^a^Median age. ^b^Mean age.

**Table 3:** Summary of meta-analysis results.

Outcomes	No. of studies	HR/RR 95% CI	*Z* value	*P* value^c^	Study heterogeneity			
					Chi^2^ (*χ* ^2^)	df	*I* ^2^, %	*P* value^c^
*Pre-DNA*								
OS	11	3.45^a^	3.23	**0.001**	42.20	10	76	**<0.00001**
PFS	5	2.37^a^	3.94	**<0.0001**	2.45	4	0	0.65
CR	3	1.45^b^	3.77	**0.0002**	0.37	2	0	0.83
ORR	5	1.57^b^	4.13	**<0.0001**	6.79	4	41	0.15
*Post-DNA*								
OS	5	2.30^a^	2.60	**0.009**	0.97	4	0	0.91
PFS	4	2.35^a^	3.14	**0.002**	2.14	3	0	0.54

No.: number; HR: hazard ratio; RR: relative risk; CI: confidence interval; pre-DNA: pretreatment EBV-DNA; post-DNA: posttreatment EBV-DNA; OS: overall survival; PFS: progression-free survival; CR: complete response; ORR: overall response rate. ^a^Hazard ratios. ^b^Risk ratios. ^c^Statistically significant results are shown in bold.

## Abbreviations

EBV: Epstein-Barr virus
CT: Chemotherapy
RT: Radiotherapy
CCRT: Concurrent chemoradiotherapy
CI: Confidence interval
OS: Overall survival
PFS: Progression-free survival
CR: Complete response
PR: Partial response
ORR: Overall response rate
HR: Hazard radio
RR: Relative risk
PCR: Polymerase chain reaction
Pre-DNA: Pretreatment EBV-DNA
Post-DNA: Posttreatment EBV-DNA
RCT: Randomized clinical trial
ENKL: Extranodal NK/T-cell lymphoma
NOS: Newcastle–Ottawa quality assessment scale.

## Supplementary Materials

Supplementary Materials Figure S1: forest plot of the analysis for pre-DNA-associated PFS. Figure S2: forest plot of the analysis for pre-DNA-associated CR. Figure S3: forest plot of the analysis for pre-DNA-associated ORR. Figure S4: forest plot of the analysis for post-DNA-associated PFS. Figure S5: sensitivity analysis. Figure S6: Begg's test of publication bias. Supplementary Table 1: results of subgroup analyses for pre-DNA-associated OS. Supplementary Table 2: results of subgroup analyses for post-DNA-associated OS.
