# Supplementary material for: The Clinical Utility of Circulating Epstein-Barr Virus DNA Concentrations in NK/T-Cell Lymphoma: A Meta-Analysis
Source: Dis Markers. 2018 Nov 18;2018:1961058. doi: 10.1155/2018/1961058 (PMC6276475; doi:10.1155/2018/1961058)
Supplement: Supplementary Materials — Figure S1: forest plot of the analysis for pre-DNA-associated PFS. Figure S2: forest plot of the analysis for pre-DNA-associated CR. Figure S3: forest plot of the analysis for pre-DNA-associated ORR. Figure S4: forest plot of the analysis for post-DNA-associated PFS. Figure S5: sensitivity analysis. Figure S6: Begg's test of publication bias. Supplementary Table 1: results of subgroup analyses for pre-DNA-associated OS. Supplementary Table 2: results of subgroup analyses for post-DNA-associated OS. [file 1961058.f1.pdf]

Figure S1 Forest plot of the analysis for pre-DNA associated PFS

Study or Subgroup	log[Hazard Ratio]	SE	Weight	Hazard Ratio IV, Fixed, 95% CI
JH Liang 2017	1.2	0.56	14.9%	3.32 [1.11, 9.95]
L Wang 2015	-0.49	1.36	2.5%	0.61 [0.04, 8.81]
QS Yang 2017	1.15	0.44	24.2%	3.16 [1.33, 7.48]
WS Liu 2015	0.82	0.32	45.7%	2.27 [1.21, 4.25]
ZY Wang 2012	0.08	0.61	12.6%	1.08 [0.33, 3.58]
**Total (95% CI)**			**100.0%**	**2.29 [1.50, 3.51]**

Heterogeneity: Chi² = 3.42, df = 4 (P = 0.49); I² = 0%
Test for overall effect: Z = 3.84 (P = 0.0001)

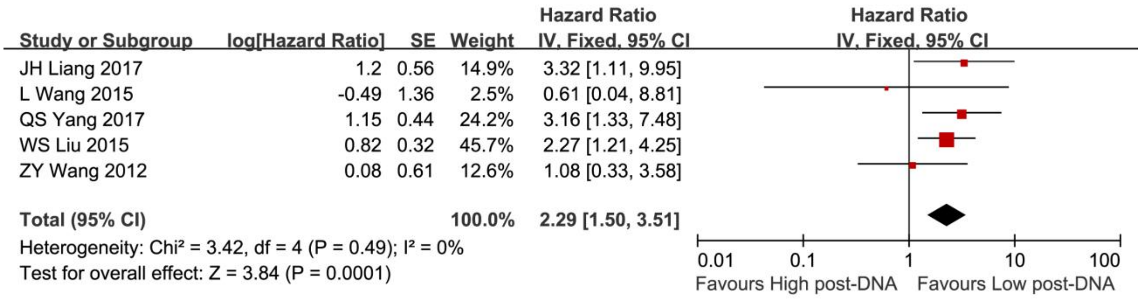

Figure S2 Forest plot of the analysis for pre-DNA associated CR

Study or Subgroup	Low pre-DNA Events	Low pre-DNA Total	High pre-DNA Events	High pre-DNA Total	Weight	Risk Ratio M-H, Fixed, 95% CI
J Zhang 2017	14	17	38	68	37.8%	1.47 [1.09, 2.00]
L Wang 2015	24	25	30	43	54.9%	1.38 [1.11, 1.70]
Y Ito 2012 (1)	10	19	2	7	7.3%	1.84 [0.53, 6.41]
**Total (95% CI)**		**61**		**118**	**100.0%**	**1.45 [1.19, 1.75]**
Total events	48		70			

Heterogeneity: Chi² = 0.37, df = 2 (P = 0.83); I² = 0%
Test for overall effect: Z = 3.77 (P = 0.0002)

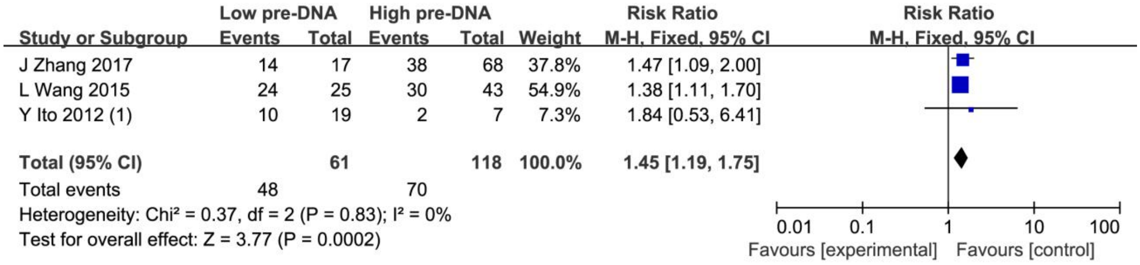

Figure S3 Forest plot of the analysis for pre-DNA associated ORR

Study or Subgroup	Low pre-DNA Events	Low pre-DNA Total	High pre-DNA Events	High pre-DNA Total	Weight	Risk Ratio M-H, Fixed, 95% CI
J Zhang 2017	16	17	48	68	34.7%	1.33 [1.10, 1.62]
JH Liang 2017	16	18	7	10	16.3%	1.27 [0.82, 1.97]
WS Liu 2015	34	56	22	53	40.8%	1.46 [1.00, 2.14]
Y Ito 2012 (1)	18	19	2	7	5.3%	3.32 [1.02, 10.75]
Y Ito 2012 (2)	19	21	1	5	2.9%	4.52 [0.78, 26.26]
**Total (95% CI)**		**131**		**143**	**100.0%**	**1.57 [1.27, 1.95]**
Total events	103		80			

Heterogeneity: Chi² = 6.79, df = 4 (P = 0.15); I² = 41%
Test for overall effect: Z = 4.13 (P < 0.0001)

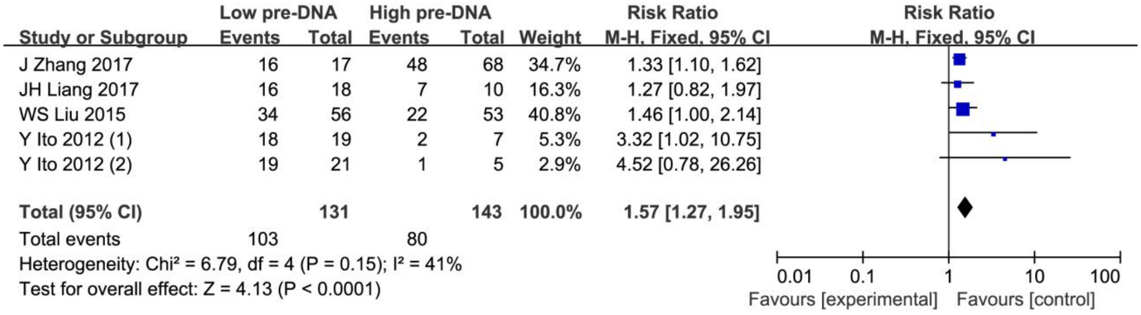

Figure S4 Forest plot of the analysis for post-DNA associated PFS

Study or Subgroup	log[Hazard Ratio]	SE	Weight	Hazard Ratio IV, Fixed, 95% CI
L Wang 2015	1.07	1.04	6.6%	2.92 [0.38, 22.38]
SH Lim 2016	0.54	0.5	28.4%	1.72 [0.64, 4.57]
SJ Kim 2015	1.28	0.41	42.3%	3.60 [1.61, 8.03]
ZY Wang 2012	0.41	0.56	22.7%	1.51 [0.50, 4.52]
**Total (95% CI)**			**100.0%**	**2.36 [1.40, 3.98]**

Heterogeneity: Chi² = 2.15, df = 3 (P = 0.54); I² = 0%
Test for overall effect: Z = 3.22 (P = 0.001)

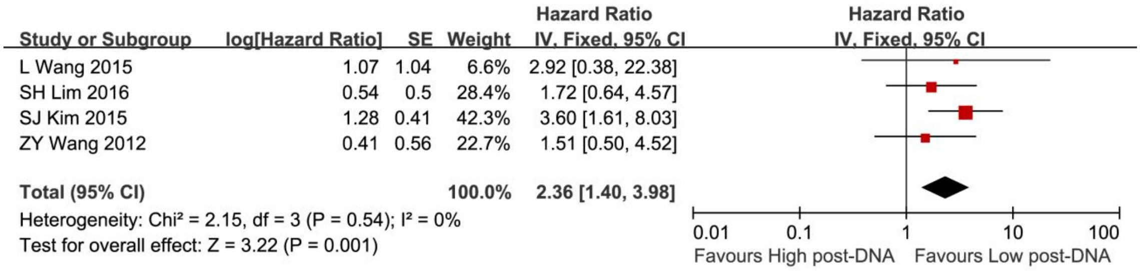

Figure S5 Sensitivity Analysis

Study or Subgroup	log[Hazard Ratio]	SE	Weight	Hazard Ratio IV, Fixed, 95% CI
HS Kim 2009	0.13	0.63	13.6%	1.14 [0.33, 3.91]
JH Liang 2017	1.36	0.57	16.6%	3.90 [1.27, 11.91]
KI Lei 2002	1.13	1.04	5.0%	3.10 [0.40, 23.77]
L Wang 2015	5.47	226.46	0.0%	237.46 [0.00, 1.376E195]
QS Yang 2017	0.65	0.47	24.4%	1.92 [0.76, 4.81]
R Liang 2016	1.25	0.61	14.5%	3.49 [1.06, 11.54]
R Suzuki 2011	2.67	1.06	4.8%	14.44 [1.81, 115.30]
Y Ito 2012 (1)	2.33	0.64	13.2%	10.28 [2.93, 36.03]
Y Ito 2012 (2)	3.97	1.12	4.3%	52.98 [5.90, 475.88]
ZY Wang 2012	0.9	1.21	3.7%	2.46 [0.23, 26.35]
**Total (95% CI)**			**100.0%**	**3.59 [2.28, 5.66]**

Heterogeneity: Chi² = 15.45, df = 9 (P = 0.08); I² = 42%

Test for overall effect: Z = 5.50 (P < 0.00001)

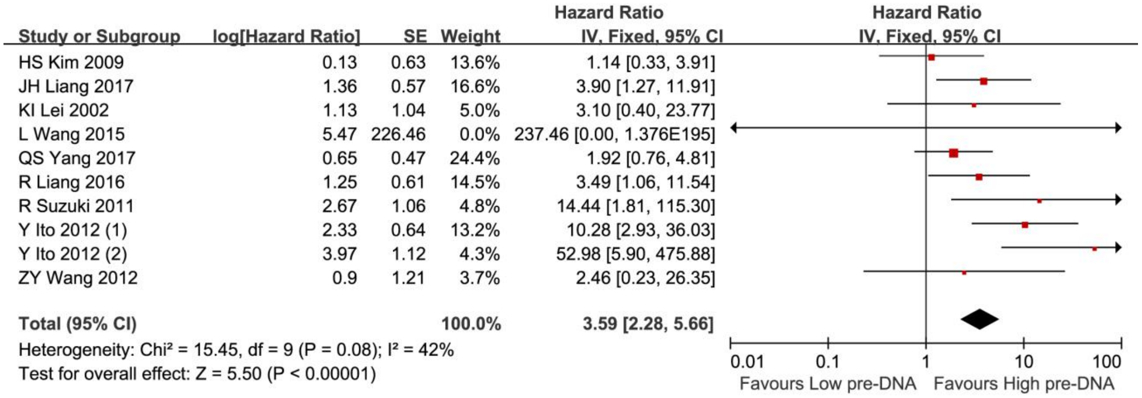

Figure S6 Begg's test of publication bias

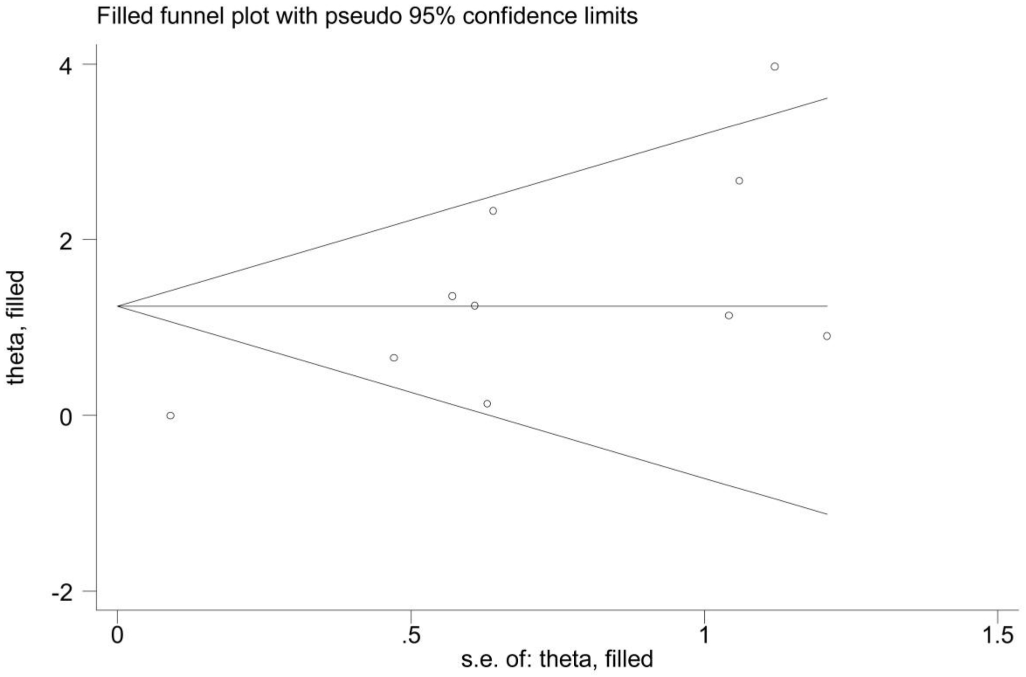

Supplementary Table 1. Results of subgroup analyses for pre-DNA associated OS

Stratified Analysis	No. of studies	No. of pts	HR (95% CI)	*P*-value[a]	Heterogeneity I^2	Heterogeneity *P*-value[a]
**Number of Participants**						
≥50	4	303	1.02 [0.86, 1.21]	0.84	0	0.49
<50	7	202	5.18 [2.28, 11.78]	**<0.0001**	53	**0.05**
**Sample of Quantification of EBV-DNA**						
plasma	8	400	3.06 [1.30, 7.16]	**0.01**	73	**0.0005**
whole blood	3	105	4.95 [0.84, 29.07]	0.08	78	**0.01**
**Ann Arbor stage**						
I-II	2	137	2.46 [0.23, 26.35]	0.46	0	0.98
III-IV	1	32	3.90 [1.27, 11.91]	**0.02**	NA	NA
I-IV	6	284	1.93 [0.98, 3.81]	0.06	62	**0.02**
**Cut-off value**						
0	2	153	0.99 [0.83, 1.18]	0.91	0	0.98
500	2	150	1.98 [0.84, 4.67]	0.12	0	0.85
others	7	202	5.18 [2.28, 11.78]	**<0.0001**	53	**0.05**

Abbreviations: No. : number; pts : patients; HR : hazard ratio; CI : confidence interval; NA : not applicable.

a Statistically significant results are shown in bold.

Supplementary Table 2. Results of subgroup analyses for post-DNA associated OS

Stratified Analysis	No. of studies	No. of pts	HR (95% CI)	*P*-value[a]	Heterogeneity	
					I^2	*P*-value[a]
**Number of Participants**						
≥50	4	293	2.77 [1.36, 5.63]	**0.005**	0	0.83
<50	1	27	1.67 [0.51, 5.40]	0.40	NA	NA
**Sample of Quantification of EBV-DNA**						
plasma	3	191	2.81 [0.92, 8.56]	0.07	0	0.65
whole blood	2	129	2.27 [1.10, 4.69]	**0.03**	0	0.51
**Ann Arbor stage**						
I-II	2	137	2.33 [0.62, 8.77]	0.21	0	0.43
I-IV	3	183	2.44 [1.23, 4.84]	**0.01**	0	0.68

Abbreviations: No. : number; pts : patients; HR : hazard ratio; CI : confidence interval; NA : not applicable.

a Statistically significant results are shown in bold.
